# Longitudinal Associations of Food Security with Health and Dietary Factors among Food FARMacy Participants during COVID-19 in New York City

**DOI:** 10.3390/nu16030434

**Published:** 2024-02-01

**Authors:** Jennifer Woo Baidal, Morgan A. Finkel, Elizabeth Kelman, Ngoc Duong, Celine Bien-Aime, Jeff Goldsmith, Sandra S. Albrecht, Emma Hulse, Alyson Rosenthal, Jeremy Reiss, Rachel Schwartz, Dodi Meyer, on behalf of the HERALD Collaborative

**Affiliations:** 1 Department of Pediatrics, Columbia University Vagelos College of Physicians & Surgeons, New York, NY 10032, USA; maf2260@cumc.columbia.edu (M.A.F.); nqd2000@cumc.columbia.edu (N.D.); cb3198@cumc.columbia.edu (C.B.-A.); ddm11@cumc.columbia.edu (D.M.); 2 NewYork-Presbyterian Morgan Stanley Children’s Hospital, New York, NY 10032, USA; 3 Department of Biostatistics, Columbia University Mailman School of Public Health, New York, NY 10032, USA; ajg2202@cumc.columbia.edu; 4 Department of Epidemiology, Columbia University Mailman School of Public Health, New York, NY 10032, USA; ssa2018@cumc.columbia.edu; 5 Division of Community and Population Health, New York Presbyterian Hospital, New York, NY 10032, USA; emh9022@nyp.org; 6 West Side Campaign Against Hunger, New York, NY 10024, USA; arosenthal@wscah.org; 7 Henry Street Settlement, New York, NY 10002, USA; jreiss@henrystreet.org; 8 Public Health Solutions, New York, NY 10013, USA; rschwartz@healthsolutions.org

**Keywords:** food pantry, food insecurity, emergency food assistance, COVID-19

## Abstract

In cross-sectional studies, food insecurity is associated with adverse health and dietary outcomes. Whether self-reported health and dietary outcomes change in response to improvements in food security has not been examined. We sought to examine how increases in food security are related to changes in health and dietary factors. In this longitudinal, observational study, we included adult participants in a clinical-community emergency food assistance program in New York City from July 2020 to November 2021. Program staff measured food security with a validated six-item measure at program enrollment and six-month re-enrollment. Participants self-reported health and dietary factors (vegetable, fruit, juice, and sugar-sweetened beverage (SSB) consumption frequency). We used multivariable regression to examine associations between change in food security with change in health and dietary factors over six months. Among 310 participants, the mean food security score improved by 1.7 ± 2.3 points over six months. In unadjusted models, each point improvement in food security was associated with increased vegetable (*β* = 0.10 times; 95% CI: 0.05–0.15); fruit (*β* = 0.08 times; 95% CI: 0.03–0.14); and juice (*β* = 0.10 times; 95% CI: 0.05–0.15) consumption. In adjusted models, results remained significant for vegetable and fruit consumption, but not juice. Change in food security was not associated with change in health or SSB outcomes. In this cohort during COVID-19, improved food security was associated with improved vegetable and fruit consumption. Randomized trials that examine the effectiveness of clinical-community partnerships focused on improving food security and nutrition are warranted.

## 1. Introduction

Food insecurity, a condition in which individuals lack reliable access to adequate, nutritious food, is a pervasive, long-standing public health issue that affected 10.5 percent of households in the United States of America (US), including 1.18 million people in New York City (NYC), in 2019 [1]. Food insecurity is associated with poor dietary quality and is increasingly recognized as an upstream factor to adverse health outcomes across the life course including obesity, cardiovascular disease, and depression [2,3,4,5,6,7,8]. Among adults, those with food insecurity have a higher risk of mortality than those without food insecurity [7,8].

Although observational studies support a link between food insecurity and adverse health outcomes, refs. [2,3,4,5,6,7,8] only one longitudinal study to date has examined how improvements in food security relate to changes in self-reported health and mental health outcomes [9]. No longitudinal research has examined whether dietary factors change in response to improvements in food security. Understanding how changes in validated food security measures relate to changes in health and diet will help identify clinically meaningful food security outcomes for use in future interventions.

New consensus recommendations support the integration of food insecurity screening into clinical care, thus enabling clinical care transformation to address food insecurity among patients [10,11,12]. However, effectiveness of clinical interventions to specifically improve food security in general patient populations is unproven [13,14,15]. Feasible approaches to link patients that are experiencing food insecurity with community-based organizations that address food insecurity must be identified to inform future research to test the effectiveness of clinically based food security interventions. Healthcare systems with integrated social needs screening and referral programs are uniquely poised to respond to food insecurity during public health emergencies. Data on the feasibility of interventions using clinical-community linkages during public health emergencies, such as the COVID-19 pandemic, will inform equitable approaches to promote food security in times of food crises. However, only two studies of food security programs linked to US healthcare systems during COVID-19 exist in the current literature [16,17,18].

The goal of this study was to examine how improvement in food security relates to change in health and dietary factors among participants in the Food FARMacy program, a multi-site clinical-community emergency food assistance program during COVID-19 delivered in NYC. We aimed to describe participants’ changes in food security, self-reported health, and dietary factors (increased vegetable and fruit; decreased juice and sugar-sweetened beverage consumption) over time, and to test the hypothesis that increased food security would be associated with improvements in health and dietary outcomes at six-month follow-up compared to baseline. Secondarily, we sought to explore whether federal supplemental nutrition program enrollment would affect associations between food security and outcomes.

## 2. Materials and Methods

### 2.1. Study Population

In this longitudinal study, we considered participants in a clinical-community emergency food assistance program (Food FARMacy) with ages ≥18 years eligible for inclusion. Those with incomplete food security responses at either baseline or 6-month follow-up were not eligible for inclusion in this analysis. NewYork-Presbyterian (NYP), an academic healthcare system in NYC affiliated with Columbia University Vagelos College of Physicians and Surgeons (Columbia) and Weill Cornell Medicine, delivered Food FARMacy. A pre-existing clinical-community partnership between the NYP Choosing Healthy and Active Lifestyles for Kids (CHALK) program, Columbia University Community Pediatrics, and West Side Campaign Against Hunger (WSCAH) using a self-selection mobile food pantry in pediatric primary care in northern Manhattan served as the foundation [19,20]. The abrupt onset of COVID-19 in New York City (NYC) led to employment and economic challenges, which resulted in sharp increases in food insecurity with 1.4 million residents in the NYC metropolitan area lacking enough food to eat in April 2020 [21]. Non-Hispanic Black and Hispanic/Latino populations were particularly impacted by COVID-19 related disparities in food security in part related to inequitable access to healthy, affordable food and other resources as a result of longstanding racist policies and practices [22,23]. Therefore, in May 2020, Columbia/NYP, WSCAH, and other community-based organizations (CBOs) launched the Food FARMacy program in response to steep increases in food insecurity during the COVID-19 pandemic in communities disproportionately burdened by food insecurity.

To facilitate physical distancing and reduce contact time with individuals, we adapted the prior self-selection model to a pre-packaged grocery distribution and delivery program. The Food FARMacy program had 17 grocery package distributions at each CBO site during the study period. Each grocery package included approximately 40 pounds of fresh fruit, vegetables, whole grains, beans, shelf-stable milk, and other groceries. Three non-profit CBOs that provide community and social services in neighborhoods served by NYP participated in Food FARMacy: CAMBA in Brooklyn, Public Health Solutions (PHS) in Queens, and Henry Street Settlement (HSS) in the Lower East Side of Manhattan.

Individuals residing in an NYP and CBO service area experiencing increased risk for food insecurity were eligible for Food FARMacy registration. Food insecurity risk was determined by response to the validated two-item Hunger Vital Signs™ Food Security Questionnaire [24,25]. Clinical staff (physicians, dietitians, social workers, and other non-provider staff) at ambulatory and inpatient sites screened patients for food insecurity and other social risks. Patients with a social risk were asked whether they would like a food assistance referral. After patients provided permission for referrals, clinical staff referred eligible individuals to the Food FARMacy program through a hand-off by phone call or email. At participating CBOs, community staff screened for social needs at the time of client intakes and connected participants to additional relevant services, including direct registration in Food FARMacy. The Columbia University Institutional Review Board approved the study with a waiver of consent because all data used in these analyses were collected for programmatic use (Columbia University IRB #AAT1498).

### 2.2. Measures

CBO staff members collected participant responses to questions about food security, health, and nutrition as part of program intake and recertification. Baseline data collection took place July 2020 to April 2021. Follow-up data collection took place January 2021 to November 2021. CBO staff measured household food security with the United States Department of Agriculture (USDA) Six-item Short Form Food Security Survey Module, with a tailored reference period of six months [26]. For each affirmative response, we assigned one point, which was in alignment with USDA technical guidance. Using this validated scoring system, responses were classified as high or marginal food security (0 or 1 points), low food security (2 to 4 points), or very low food security (5 or 6 points). We classified the presence of household food insecurity as those with low or very low food security [26]. For ease of results interpretation, we reverse-coded the total raw food security score so that a higher score represented higher food security. Hereafter, we refer to the food security score as the reverse-coded score where a score of six corresponds to food security.

Food FARMacy participants responded to a one-item question about self-reported health at baseline and follow-up [27,28]. Response options included excellent, very good, good, fair, and poor. Self-reported health was dichotomized at each time point in alignment with standard reporting conventions (excellent/very good/good vs. fair/poor) [29]. To create change scores for regression analyses, we calculated the difference between baseline and follow-up self-reported health using a continuous measure ranging from 5 (excellent) to 1 (poor).

For dietary factors at baseline and follow-up, participants verbally responded to four questions about their vegetable, fruit, juice, and sugar-sweetened beverage consumption from the School Physical Activity and Nutrition (SPAN) monitoring system [30]. The SPAN questions asked about behaviors yesterday with a 4-point response scale (e.g., 0 = did not eat any vegetables, 1 = ate vegetables 1 time yesterday; 2 = ate vegetables 2 times yesterday; 3 = ate vegetables 3 or more times yesterday). To create change scores for regression analyses, we considered responses of three or more times equal to three because a small proportion of individuals had responses of 2 times or more.

At baseline, participants answered questions about characteristics such as age, gender, race, ethnicity, household income, and household size. We considered race and ethnicity as confounders via structural injustices, not biological mechanisms. Race and ethnicity were combined into one variable and categorized as Hispanic/Latino regardless of race, non-Hispanic Black, non-Hispanic other (including white), or missing.

At baseline and follow-up, participants reported household enrollment in WIC or SNAP. Because WIC and SNAP enrollment were similar at each time point, we dichotomized WIC/SNAP enrollment based on the report of household member enrollment in WIC, SNAP, or both at either time point.

### 2.3. Statistical Analysis

We used descriptive statistics to examine baseline participant characteristics by food security status and distributions of outcomes. To examine the overall change over the six-month period for food security, we used the McNemar–Bowker test for categorical variables with more than two levels (three-level food security status), and the paired Wilcoxon Signed Rank test was used for continuous variables (food security score). We used McNemar’s Chi-squared test to examine difference between baseline and six-month follow-up in health and dietary factors as dichotomous variables.

In unadjusted and multivariable linear regression models, we examined associations of food security score change with self-reported health and dietary outcome changes. In multivariable regression models, we first adjusted for participant covariates (participant age, household income, and household size). Most participants reported female gender, thus gender was not included as a potential confounder. Participant education and insurance type were included in data collection and reported in demographic characteristics but were not considered as confounders because of their potential mediating effects between food security and outcomes. Contextual covariates were included in two models as potential confounders. First, in the site-adjusted model, program site was added to the individual-level model. Second, a race/ethnicity-adjusted model was created by additionally adjusting the site-adjusted model for participant race/ethnicity. The race/ethnicity model was a priori considered the final multivariable-adjusted model. To explore whether WIC/SNAP participation status modified effect estimates, WIC/SNAP enrollment was additionally adjusted for in regression models and a two-way interaction term for food security change and WIC/SNAP enrollment was tested with each outcome. All hypothesis tests were two-sided, with statistical significance defined as *p* < 0.05 for regression models. For two-way interaction terms, p-interaction < 0.10 was considered statistically significant. Data analysis took place from December 2021 to September 2022 using R Statistical Software (version 4.1.1).

## 3. Results

A total of 492 participants registered in the Food FARMacy program during the study period (Supplementary Figure S1). At six-month follow-up, 310 had complete baseline and follow-up food security responses and comprise the analytic sample.

Overall, participant mean age was 45.9 ± standard deviation (SD) 14.6 years (Table 1). Most participants identified as female (89%) and Hispanic/Latino (65%). Most eligible participants were enrolled in Medicaid and/or Medicare, and the group missing six-month follow-up data had lower proportion of Hispanic/Latino ethnicity than those with complete data at follow-up (Supplementary Table S1). Otherwise, those with six-month follow-up responses had similar age, gender, household income, household size, and food security to those without complete follow-up responses.

**Figure 1:**
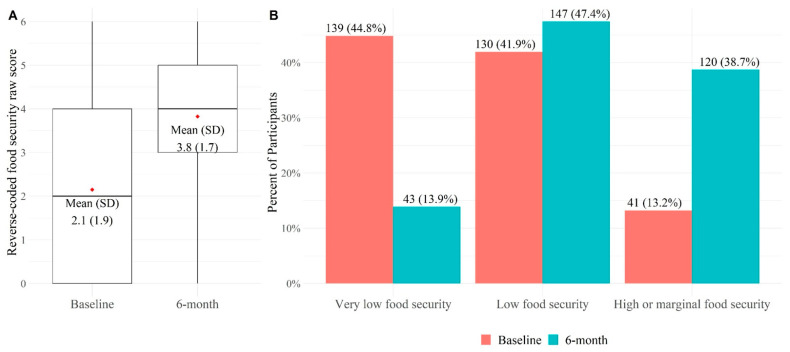
Baseline and 6-month Food Security among 310 Food FARMacy Participants. (**A**) The boxplot illustrates the distribution of the reverse coded food security raw score in the analytical sample at baseline and follow-up. The box represents the interquartile range (25th–75th percentile), the line within the box marks the median, and the whiskers represents the range of the data. Wilcoxon signed-rank test was used to compare food security score (reverse-coded score where 6 is food secure) at baseline to 6 months (*p* < 0.001). Mean food security score difference from baseline to 6 months was 1.7 ± 2.3 points; (**B**) McNemar–Bowker test was used to compare the proportions of food security categories at baseline to 6 months (*p* < 0.001).

Overall, the mean food security score improved from 2.1 ± SD 1.9 at baseline to 3.8 ± SD 1.7 at follow-up (*p* < 0.001, Figure 1). The proportion of participants with high/marginal food security increased (13% at baseline, 39% at follow-up, *p* < 0.001) and those with very low food security decreased (45% at baseline to 14% at follow-up, *p* < 0.001) over 6 months.

The proportion of individuals who self-rated health as excellent, very good, or good increased (Table 2). Most individuals reported consuming items zero or one times the day prior for all categories; thus, we examined dichotomous data for change in health and dietary outcomes among the overall cohort. Those who ate any vegetables, ate any fruit, and did not consume any sugar-sweetened beverages the day prior increased from baseline to follow-up. The proportion that drank any juice increased. All results were statistically significant.

In regression models, we found that each point improvement in food security was associated with a small increase in the frequency of vegetable intake [*β* = 0.10 times yesterday (95% CI: 0.05, 0.15 times)] in the unadjusted model (Table 3). Results were similar after adjusting for participant characteristics but were attenuated after additionally adjusting for site [*β* = 0.06 times yesterday (95% CI: 0.01, 0.12 times)]. Results were not further attenuated after adjusting for race/ethnicity. For fruit intake, increased food security was associated with increased frequency of fruit intake in unadjusted models [*β* = 0.08 times yesterday (95% CI: 0.03, 0.14 times)] and estimates remained similar in all adjusted models. For juice, increased food security was associated with increased frequency of juice consumption in unadjusted, participant-adjusted, and site-adjusted models. After additionally adjusting for race/ethnicity, associations between change in food security and change in juice consumption remained similar in direction, but confidence intervals crossed zero. We did not find statistically significant associations of change in food security with change in self-rated health or change in SSB consumption in any models. In models exploring whether additionally adjusting for WIC/SNAP enrollment modified associations between change in food security and outcomes, effect estimates and confidence intervals did not substantially change from the final race/ethnicity-adjusted model for all outcomes and all p-interaction terms were >0.10.

## 4. Discussion

In this longitudinal study of a clinical-community emergency food assistance program during COVID-19, participants experienced improvements in household food security, self-reported health, fruit intake, vegetable intake, and sugar-sweetened beverage consumption over 6 months. Improvements in food security were associated with statistically significant improvements in the frequency of vegetable and fruit consumption. Our findings are the first to support that an improvement in food security is linked to increased frequency of vegetable and fruit consumption, thus supporting the use of food security outcomes in future interventions to reduce diet-related chronic diseases.

Expert consensus recommendations and new requirements include clinical screening for social needs such as household food insecurity [10,11,12,31]. However, clinically meaningful endpoints for food security interventions remain undefined. In a recently published retrospective longitudinal study by Berkowitz et al. using data from 2016–2017 among adults with food insecurity, a 1-point increase in food security on a 10-item scale was associated with a 0.38-point improvement in mental health, a 0.15-point improvement in psychological distress, a 0.05-point improvement in depressive symptoms, and a 0.003-point improvement in health utility over one year [9]. In the current study, each one-point increase in food security on a six-item scale was associated with increases in frequency of fruit and vegetable consumption. These two studies support the use of food security as a research outcome measure. Future research should delineate measures to use in specific clinical settings for screening and intervention response across life course periods.

Most Food FARMacy participants reported improved but persistent food insecurity at 6-month follow-up despite twice monthly grocery distribution, enrollment in WIC and/or SNAP, and ability to use other food programs and pantries. Among New York City residents, inability to buy groceries because of lack of money was estimated at 21–23% prevalence in April–October 2020 [23]. In that survey, respondents reported that food availability had improved by October 2020, but inadequate household income persisted as a barrier to sufficient food [23]. In a systematic review of food insecurity during the COVID-19 pandemic using data from multiple countries, increased food insecurity and decreased food availability was reported as due to insufficient income and savings [32]. In the current study, using the USDA Six-item Food Security Module among people presenting for enrollment in an emergency food assistance program, household food insecurity decreased from 88% at baseline to 61% at 6-month follow-up. Prior research suggests that food insecurity can persist despite enrollment in nutrition programs such as WIC and SNAP because of benefits inadequacy, high food costs, and barriers to continuous enrollment, and because by definition, these programs are meant to be supplemental in nature [33,34,35,36,37]. Contrary to our hypothesis that WIC/SNAP enrollment would increase associations of improved food security with health and dietary outcomes, we did not find evidence that WIC/SNAP enrollment modified the current results.

Most existing food insecurity interventions in clinical settings focus on adult caretakers of children, uninsured adults, or adults with specific chronic diseases such as cancer or type 2 diabetes to improve treatment adherence or specific clinical outcomes [13,14,15]. The concept of Food is Medicine is gaining increasing attention as a way to prevent and treat disease [38]. In a recent pilot randomized controlled trial (RCT) of a fruit and vegetable prescription program among a diverse population of adult patients with uncontrolled type 2 diabetes experiencing low incomes, preliminary evidence for decreased hemoglobin A1C within the treatment group was found, but food security and dietary outcomes were not reported [39]. In an RCT of a community-supported agriculture (CSA) intervention, dietary quality as measured by the Healthy Eating Index 2010 and food security improved among overweight or obese adult patients [40]. In that study, most participants reported non-Hispanic white race/ethnicity and lived in less urban communities compared to the Hispanic/Latino and non-Hispanic Black urban-dwelling population in the current study [40]. In contrast to these studies focused on produce provision, the current study included participants in a program where they receive all food groups. In the current study, rather than targeting a specific disease, we included those with food security screening responses that indicated risk for food insecurity across wide age ranges to focus on improving food security to promote health and disease prevention. Our study is one of the first to report individual-level longitudinal changes in food security among participants in a clinically based emergency food assistance program after the onset of the COVID-19 pandemic. Our results show that health systems can rapidly mobilize to partner with community-based organizations to address food insecurity during public health crises. Further understanding of the types and amounts of groceries needed to meaningfully improve food security, health, and nutrition in contemporary circumstances using randomized study designs among patients are needed for longer term improvements in food security outside of public health emergencies.

The 2022 White House National Strategy on Hunger, Nutrition, and Health set a goal to cut the number of households with food insecurity in half by 2030 [41,42]. In our study, this goal was not met because 269 participants had food insecurity at baseline and 187 had food insecurity at follow-up despite provision of a large amount of groceries twice monthly. Our results show that the complexity of achieving food security reaches beyond provision of food and nutrition education. Clinical-community interventions may provide timely emergency food support for households experiencing food insecurity. However, longer-term cross-sector investments are needed to reduce poverty, holistically address social needs, provide equitable education and employment opportunities, and strengthen food systems to meet goals set forth by the White House to end hunger and improve nutrition and health equity [41]. As climate change, inflation, and disruptions to food supply chains are likely to present future shocks and stressors to food systems [43], understanding clinical-community interventions to improve food security and nutrition during the COVID-19 pandemic will help inform food system responses to future emergencies.

## 5. Limitations

This observational, longitudinal study has several limitations. Developing a comparison group was not possible nor ethical during an acute food crisis, greatly limiting causal inference. The role of other changes related to COVID-19 such as food availability, food price changes, employment fluctuations, and government benefits receipt (e.g., universal school meals, expanded SNAP benefits, child tax credits) cannot be examined in the current study. However, related qualitative results reported elsewhere suggest that participants perceived that the Food FARMacy program played a critical role in continued access to fruits, vegetables, and food staples to impact their diets during COVID-19 [44]. The results are specific to Food FARMacy participants in New York City during COVID-19 and may not be generalizable to other settings. All outcomes were self-reported and may be subject to social desirability bias.

## 6. Conclusions

Clinical-community partnerships to address food insecurity during public health crises are feasible. In a cohort of adult New Yorkers participating in a clinical-community emergency food assistance program developed in response to COVID-19, participants experienced improvements in self-reported household food security, health, and dietary factors over six months. Longitudinal improvements in food security were associated with improvements in vegetable and fruit consumption, supporting the use of food security as an outcome in future clinical trials. Randomized clinical trials to improve food security as a means to improve nutrition and downstream chronic disease are warranted.

## Figures and Tables

**Table 1:** Baseline Characteristics among Food FARMacy Participants According to Baseline Food Security ^a^.

	Overall (*n* = 310)	Very Low Food Security (*n* = 139)	Low Food Security (*n* = 130)	High/Marginal Food Security (*n* = 41)
Baseline Characteristic				
Age at visit (year), mean ± SD ^b^	45.9 ± 14.6	47.8 ± 15.8	44.9 ± 14.4	43.0 ± 9.6
Female, *n* (%)	275 (88.7)	123 (88.5)	117 (90.0)	35 (85.4)
Race/Ethnicity, *n* (%)				
Hispanic/Latino	201(64.8)	87 (62.6)	86 (66.2)	28 (68.3)
Non-Hispanic, Black	69 (22.3)	42 (30.2)	22 (16.9)	5 (12.2)
Non-Hispanic, Other	29 (9.4)	7 (5.0)	15 (11.5)	7 (17.1)
Missing	11 (3.5)	3 (2.2)	7 (5.4)	1 (2.4)
Household size, mean ± SD ^b^	3.8 ± 1.8	3.5 ± 1.9	3.8 ± 1.7	4.7 ± 1.7
Missing	3	1	2	0
Annual household income, *n* (%)				
<$10,000	105 (33.9)	56 (40.3)	41 (31.5)	8 (19.5)
$10,000–$20,000	81 (26.1)	38 (27.3)	35 (26.9)	8 (19.5)
>$20,000–$35,000	74 (23.9)	24 (17.3)	30 (23.1)	20 (48.8)
Missing	50 (16.1)	21 (15.1)	24 (18.5)	5 (12.2)
Site, *n* (%)				
A	61 (19.7)	24 (17.3)	32 (24.6)	5 (12.2)
B	147 (47.4)	41 (29.5)	73 (56.2)	33 (80.5)
C	102 (32.9)	74 (53.2)	25 (19.2)	3 (7.3)
WIC and/or SNAP ^c^ *n* (%)				
Currently enrolled	184 (59.4)	68 (48.9)	88 (67.7)	28 (68.3)
Not currently enrolled	105 (33.9)	61 (43.9)	32 (24.6)	12 (29.3)
Missing	21 (6.8)	10 (7.2)	10 (7.7)	1 (2.4)

^a^ Food Security measured using US Department of Agriculture Six-item Short Form Food Security Module [26]. ^b^ Standard deviation; ^c^ WIC: Women, Infants, and Children; SNAP: Supplemental Nutritional Assistance Program.

**Table 2:** Self-Reported Health and Dietary Outcomes Among 310 Food FARMacy Participants ^a^.

	Baseline	6-Month	*p*-Value ^b,c^
Outcome, *n* (%)			
Self-reported health (*n* = 302)			0.011 *
Excellent, Very good, Good	159 (52.6)	183 (60.6)	
Fair, Poor	143 (47.4)	119 (39.4)	
Vegetable consumption yesterday (*n* = 310)			<0.001 ***
0 times	102 (32.9)	41 (13.2)	
1 or more times	208 (67.1)	269 (86.8)	
Fruit consumption yesterday (*n* = 307)			<0.001 ***
0 times	114 (37.1)	49 (16.0)	
1 or more times	193 (62.9)	258 (84.0)	
Juice consumption yesterday (*n* = 310)			0.025 *
0 times	195 (62.9)	170 (54.8)	
1 or more times	115 (37.1)	140 (45.2)	
SSB consumption (*n* = 307) ^d^			<0.001 ***
0 times	149 (48.5)	220 (71.7)	
1 or more times	158 (51.5)	87 (28.3)	

^a^ Dietary factors measured using the elementary school School Physical Activity and Nutrition Survey [30]. ^b^ McNemar’s Chi-squared test. ^c^ Boldface indicates statistical significance (* *p* < 0.05, *** *p* < 0.001). ^d^ SSB, sugar-sweetened beverage.

**Table 3:** Longitudinal Associations of Food Security with Health and Dietary Outcomes among 310 Participants.

	Unadjusted Model ^a^	Participant-Adjusted Model ^b^	Site-Adjusted Model ^c^	Race/Ethnicity-Adjusted Model ^d^	WIC/SNAP ^e^-Adjusted Model ^f^
	Mean Difference per Point Improvement in Food Security (95% CI)				
Change in Continuous Outcomes ^g^					
Self-Rated Health, points	0.03 (−0.01, 0.08)	0.04 (−0.01, 0.09)	0.02 (−0.03, 0.08)	0.02 (−0.04, 0.07)	0.02 (−0.04, 0.08)
Vegetable intake, times yesterday	0.10 ** (0.05, 0.15)	0.10 ** (0.05, 0.15)	0.06 * (0.01, 0.12)	0.06 * (0.003, 0.11)	0.06 * (0.002, 0.11)
Fruit intake, times yesterday	0.08 ** (0.03, 0.14)	0.09 ** (0.04, 0.14)	0.07 * (0.01, 0.13)	0.07 * (0.01, 0.13)	0.07 * (0.01, 0.13)
Juice consumption, times yesterday	0.10 ** (0.05, 0.15)	0.09 ** (0.04, 0.15)	0.07 * (0.01, 0.13)	0.05 (−0.01, 0.11)	0.05 (−0.01, 0.11)
SSB ^h^ consumption, times yesterday	−0.03 (−0.08, 0.02)	−0.03 (−0.08, 0.02)	−0.05 (−0.11, 0.01)	−0.05 (−0.11, 0.02)	−0.05 (−0.11, 0.02)

^a^ Linear regression model with food security score change as main predictor; ^b^ Participant-adjusted model: adjusted for participant age, household income, and household size; ^c^ Site-adjusted model: individual-adjusted model additionally adjusted for site as a proxy for neighborhood factors; ^d^ Race/ethnicity-adjusted model: site-adjusted model additionally adjusted for race/ethnicity; ^e^ WIC: Women, Infants, and Children; SNAP: Supplemental Nutrition Assistance Program; ^f^ WIC/SNAP-adjusted model: race/ethnicity-adjusted model additionally adjusted for enrollment in WIC and/or SNAP at either baseline or follow-up; ^g^ Dietary factors measured using the elementary school School Physical Activity and Nutrition Survey [30]; ^h^ SSB: Sugar-sweetened beverage; * *p* < 0.05, ** *p* < 0.01.

## Data Availability

Dataset available on request from the authors.

## Supplementary Materials

The following supporting information can be downloaded at: https://www.mdpi.com/article/10.3390/nu16030434/s1, Figure S1: Participant Eligibility and Inclusion in Single-arm Study of Food FARMacy Participants in New York City; Table S1: Baseline Characteristics According to Food Security Data Completion.
