# Supplementary material for: Longitudinal Associations of Food Security with Health and Dietary Factors among Food FARMacy Participants during COVID-19 in New York City
Source: Nutrients. 2024 Feb 1;16(3):434. doi: 10.3390/nu16030434 (PMC10857290; doi:10.3390/nu16030434)
Supplement: Supplementary file 1 [file nutrients-16-00434-s001.zip › nutrients-2800171-supplementary.pdf]

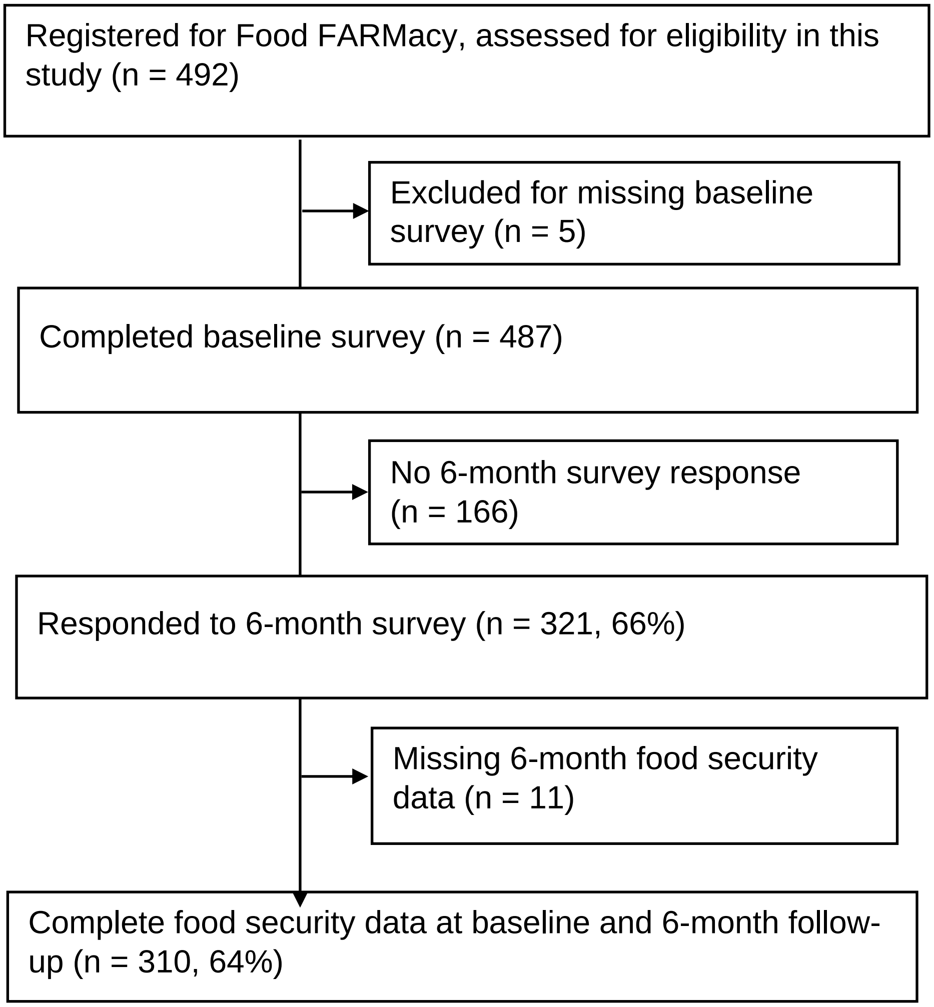

Figure S1. Participant Eligibility and Inclusion in Single-arm Study of Food FARMacy Participants in New York City.

Table S1. Baseline Characteristics According to Food Security Data Completion.

	**Overall (N = 487)**	**Both timepoints (N = 310)**	**Baseline only (N = 177)**
**Baseline Characteristic**			
Age at enrollment (year), mean ± SD	45.6 ±15.0	45.9 ±14.6	45.1 ±15.8
Female, n (%)	427 (87.7)	275 (88.7)	152 (85.9)
Race/Ethnicity, n (%)			
Hispanic/Latino	298 (61.2)	201 (64.8)	97 (54.8)
Non-Hispanic, Black	126 (25.9)	69 (22.8)	57 (32.2)
Non-Hispanic, Other	48 (9.8)	29 (9.4)	19 (10.7)
Missing	15 (3.1)	11 (3.5)	4 (2.3)
Household size, mean ± SD	3.7 ± 1.8	3.8 ± 1.8	3.5 ± 1.8
Missing	5	3	2
Annual household income, n (%)			
<$10,000	170 (34.9)	105 (33.9)	65 (36.7)
$10,000-$20,000	125 (25.7)	81 (26.1)	44 (24.9)
>$20,000	116 (23.8)	74 (23.9)	42 (23.7)
Missing	76 (15.6)	50 (16.1)	26 (14.7)
Site, n (%)			
A	93 (19.1)	61 (19.7)	32 (18.1)
B	223 (45.8)	147 (47.4)	76 (42.9)
C	171 (35.1)	102 (32.9)	69 (39.0)
WIC/SNAP [a] participation, n (%)			
Receiving both	59 (12.1)	40 (12.9)	19 (10.7)
Receiving either	219 (45.0)	144 (46.5)	75 (42.4)
Receiving none	175 (35.9)	105 (33.9)	70 (39.5)
Missing	34 (7.0)	21 (6.8)	13 (7.3)
Highest education, n (%)			
High school or below	324 (66.5)	209 (67.4)	115 (65.0)
Some college or higher	158 (32.4)	97 (31.3)	61 (34.5)
Missing	5 (1.0)	4 (1.3)	1 (0.5)
Health Insurance, n (%)			
Medicare only	9 (1.8)	4 (1.3)	5 (2.8)
Medicaid only	239 (49.1)	149 (48.1)	90 (50.8)
Both Medicaid and Medicare	81 (16.6)	58 (18.7)	23 (13.0)
Other	21 (4.3)	13 (4.2)	8 (4.5)
None	91 (18.7)	55 (17.7)	36 (20.3)
Missing	46 (9.4)	31 (10.0)	15 (8.5)
Food Security Raw Score,[b] mean ± SD	3.90 ± 1.88	3.85 ± 1.90	3.97 ± 1.84
Reverse-coded Food Security, mean ± SD	2.10 ± 1.88	2.15 ± 1.90	2.03 ± 1.84
Household Food Security,[b] n (%)			
High or Marginal	59 (12.1)	41 (13.2)	18 (10.2)
Very low or Low (food insecure)	428 (87.9)	269 (86.8)	159 (89.8)
Low security	203 (41.7)	130 (41.9)	73 (41.2)
Very low security	225 (46.2)	139 (44.9)	86 (48.6)

[a]WIC: Women, Infants, and Children; SNAP: Supplemental Nutritional Assistance Program
[b]Food Security measured using US Department of Agriculture Six-item Short Form Food Security Module.[18]
